# Acyl–Acyl Carrier Protein Desaturases and Plant Biotic Interactions

**DOI:** 10.3390/cells10030674

**Published:** 2021-03-18

**Authors:** Sami Kazaz, Romane Miray, Sébastien Baud

**Affiliations:** Institut Jean-Pierre Bourgin, INRAE, CNRS, AgroParisTech, Université Paris-Saclay, 78000 Versailles, France; sami.kazaz@inrae.fr (S.K.); romane.miray@inrae.fr (R.M.)

**Keywords:** acyl–acyl carrier protein desaturase, fatty acid, monounsaturated, plant, biotic interactions

## Abstract

Interactions between land plants and other organisms such as pathogens, pollinators, or symbionts usually involve a variety of specialized effectors participating in complex cross-talks between organisms. Fatty acids and their lipid derivatives play important roles in these biological interactions. While the transcriptional regulation of genes encoding acyl–acyl carrier protein (ACP) desaturases appears to be largely responsive to biotic stress, the different monounsaturated fatty acids produced by these enzymes were shown to take active part in plant biotic interactions and were assigned with specific functions intrinsically linked to the position of the carbon–carbon double bond within their acyl chain. For example, oleic acid, an omega-9 monounsaturated fatty acid produced by Δ^9^-stearoyl–ACP desaturases, participates in signal transduction pathways affecting plant immunity against pathogen infection. Myristoleic acid, an omega-5 monounsaturated fatty acid produced by Δ^9^-myristoyl–ACP desaturases, serves as a precursor for the biosynthesis of omega-5 anacardic acids that are active biocides against pests. Finally, different types of monounsaturated fatty acids synthesized in the labellum of orchids are used for the production of a variety of alkenes participating in the chemistry of sexual deception, hence favoring plant pollination by hymenopterans.

## 1. Introduction

In plant cells, fatty acids produced in the plastids are used for the biosynthesis of a variety of lipid compounds. Acyl lipids derived from fatty acids are basic structural components of the cellular membranes [1]. Triacylglycerols represent an important carbon and energy store in many oleaginous species [2,3]. Cuticular lipids such as cutin, suberin, and cuticular waxes constitute lipophilic cell-wall associated barriers [4]. Aside from this, some fatty acids, acyl lipids, and their derivatives participate in signal transduction pathways that influence plant development and responses to environmental cues [5,6]. These various categories of lipids play fundamental roles in biotic interactions. First, cuticular lipid layers, together with cell walls, can serve as structural obstruction and limit the entry of pathogenic microorganisms. Changes in cuticular permeability have thus been associated with altered plant responses to microbes [7,8,9]. Early in plant–pathogen interactions, plants recognize specific molecular signatures in microbial cells called pathogen-associated molecular patterns (PAMPs) via cell surface-localized pattern recognition receptors. This recognition results in activation of PAMP-triggered immunity (PTI), a form of basal resistance minimizing or preventing microbial colonization [10]. Before and concurrent with the onset of PTI, changes in membrane lipid composition and adjustment of membrane fluidity largely mediated by desaturases appear to be critical for the function of integral membrane proteins that participate in this response [11,12]. Virulent pathogens can overcome PTI by deploying virulence effectors that interfere with components of the PTI transduction pathway. In response, plants have another induced defense response called effector-triggered immunity (ETI), which is induced upon recognition of pathogen-encoded avirulence factors by the products of plant resistance (R) genes. ETI leads to the activation of a signal transduction pathway resulting in the hypersensitive response (HR) and systemic acquired resistance (SAR), two important components of a plant’s defense arsenal against pathogens [13]. The HR is a rapid response characterized by the production of reactive oxygen species (ROS) referred to as the oxidative burst, which prevents further spread of the pathogen, and by localized programmed cell death [14]. Concurrent with the HR, a systemic signal inducing long-lasting SAR in uninfected plant tissues is released. ETI shares many signaling components with PTI, but results in much stronger and more durable resistance. Enzymatic and non-enzymatic genesis of bioactive lipid mediators such as oxylipins, jasmonic acid, or azelaic acid, among others, play a key role in the deployment of plant immune responses leading to genome-wide massive transcriptional reprogramming [10]. As for storage lipids, the fatty acid composition of oilseeds is regarded as a component of pathogen susceptibility and seed colonization in some species. For example, colonization of *Glycine max* (soybean) seeds by *Cercospora kikuchii* has been found to be correlated with the ω-9 18:1 (oleic acid):18:2 (linoleic acid) ratio and mid-oleic genotypes are more extensively colonized by the fungal pathogen in the field [15].

Biosynthesis of fatty acids and their derivatives therefore play a pivotal role in plant biotic interactions, and the importance of unsaturated fatty acids is increasingly documented [12]. This review aims to summarize how plant monounsaturated fatty acids and their biosynthesis by acyl–acyl carrier protein (ACP) desaturases (AADs) contribute to plant defenses against pathogens and also participate in various plant–insect interactions. Stimulating discoveries in this research field have improved the basics of our understanding of plant–pathogen interactions. They have also impacted the improvement of agronomic varieties with respect to stress resistance. For example, ω-7 16:1 (palmitoleic acid) has been shown to directly inhibit the growth of the fungus *Verticillium dahlia*. Transgenic *Solanum Melongena* (eggplant) lines expressing the yeast Δ^9^ desaturase-coding gene *ELO1* and consequently accumulating increased levels of ω-7 16:1 display improved resistance to the fungus [16]. Similarly, expression of the yeast *ELO1* gene in *Solanum esculentum* (tomato) improves resistance to *Erysiphe polygoni* (powdery mildew) [17].

## 2. Synthesis of Monounsaturated Fatty Acids by Acyl–Acyl Carrier Protein (ACP) Desaturases

Plant de novo fatty acid synthesis occurs in plastids [18,19]. The first committed step for this pathway consists in the ATP-dependent formation of malonyl-CoA from acetyl-CoA and bicarbonate through the action of acetyl–CoA carboxylase [20]. The malonyl group of malonyl–CoA is then transferred to a protein cofactor named ACP by a malonyl-CoA:ACP S-malonyltransferase. The malonyl-ACP thus formed provides two-carbon units for each cycle of the fatty acid biosynthetic process catalyzed by a type II fatty acid synthase, which uses acetyl–CoA as a starting unit and malonyl–ACP as the elongator [21]. The fatty acid synthase complex associates four monofunctional enzymes: a 3-ketoacyl–ACP synthase catalyzing a condensation reaction, a 3-ketoacyl–ACP reductase catalyzing a first reduction step, a hydroxyacyl–ACP dehydratase catalyzing a dehydration reaction, and an enoyl–ACP reductase catalyzing a second reduction step yielding saturated acyl chains [22,23,24]. A fraction of these saturated acyl chains is hydrolyzed by acyl–ACP thioesterases and directly used for the elaboration of different classes of lipids inside and outside the plastids [25]. However, stromal AADs efficiently introduce carbon–carbon double bonds (also called unsaturations) within these saturated acyl chains to form *cis*-monounsaturated fatty acyl chains before their hydrolysis by thioesterases [26].

Δ^9^-Stearoyl–ACP desaturases represent the predominant AAD isoforms in most land plants. They efficiently desaturate 18:0 (stearic acid) to form ω-9 18:1, a major monounsaturated fatty acid of membrane and storage lipids [27]. Once esterified to a glycerol backbone to form membrane lipids, oleic acid can be further desaturated by membrane-bound fatty acid desaturases (FADs), yielding polyunsaturated fatty acids like 18:2 and 18:3 (alpha-linolenic acid). While archetypal Δ^9^-stearoyl–ACP desaturases can be found in every land plant, plant genomes usually code multiple AAD isoforms. Some of these isoforms can exhibit different substrate specificities or regioselectivities associated with the biosynthesis of unusual monounsaturated fatty acids. Δ^9^-palmitoyl–ACP desaturases identified in several plant species prefer 16:0 (palmitic acid) instead of 18:0 as a substrate, resulting in the production of ω-7 16:1 [28,29,30]. The Δ^4^- and Δ^6^-palmitoyl–ACP desaturases respectively identified in *Coriandrum sativum* (coriander) and *Thunbergia alata* (black-eyed Susan vine) also use 16:0 as a preferential substrate, but differ in their regiospecificity, leading to the production of ω-12 16:1 (Δ^4^ hexadecanoic acid) and ω-10 16:1 (Δ^6^ hexadecenoic acid) [31,32].

AADs perform dioxygen-dependent dehydrogenation reactions resulting in the introduction of double bonds into fatty acyl chains [33,34,35]. These reactions are initiated by the energy-demanding abstraction of a hydrogen from a methylene group with the use of an active-site diiron cluster recruiting and activating molecular oxygen [36]. The reducing equivalents needed for the reaction in the form of NADPH are transferred from ferredoxin reductase to ferredoxin, and then to AAD [37]. Crystal structures of *Ricinus communis* (castor) Δ^9^-stearoyl–ACP desaturase [38] and *Hedera helix* (English ivy) Δ^4^-palmitoyl–ACP desaturase [39] showed the AADs to be homodimeric proteins. Each monomer is folded into a compact single domain. The diiron active site is buried within a conserved four-helix bundle at the core of the monomer. This active site is positioned alongside a deep, narrow hydrophobic cavity [35]. The methyl end of a saturated fatty acyl chain bound to ACP can enter this hydrophobic cavity and, once this substrate is accommodated, a *cis* double bond is created between two adjacent carbon atoms of the acyl chain facing the active site, concomitantly with the reduction of a dioxygen molecule into water [40]. The boomerang-like shape of the hydrophobic channel imparts an eclipsed substrate conformation, leading to the formation of a *cis* double bond and explains the mechanism of stereoselectivity [41]. The structural basis for the different chain-length and double bond positional specificities of AADs were identified through the characterization of desaturases exhibiting different functional properties despite sharing high amino acid sequence similarity [42]. In this respect, approaches of site-directed mutagenesis aimed at converting the activity of one type of AAD to that of another by replacing specific residues have been proven to be very informative [43]. First, the conversion of a Δ^9^-stearoyl-ACP desaturase into a Δ^9^-palmitoyl-ACP desaturase was made possible by substituting amino acid residues lining the bottom part of the substrate pocket. A group of eight residues was shown to set constraints on the chain lengths of fatty acid substrates, thus determining the substrate specificity of the enzymes [28,30,43]. Then, determination of crystal structures of AADs in complex with acyl-ACPs identified residues at the entrance of the substrate-binding cavity that are determinants for the binding modes of ACP with respect to the desaturase, predisposing the potential insertion depth of the acyl chain and thereby influencing regioselectivity [44].

## 3. Transcriptional Responses of *AAD* Genes to Biotic Stress in Arabidopsis Leaves

**Figure 1:**
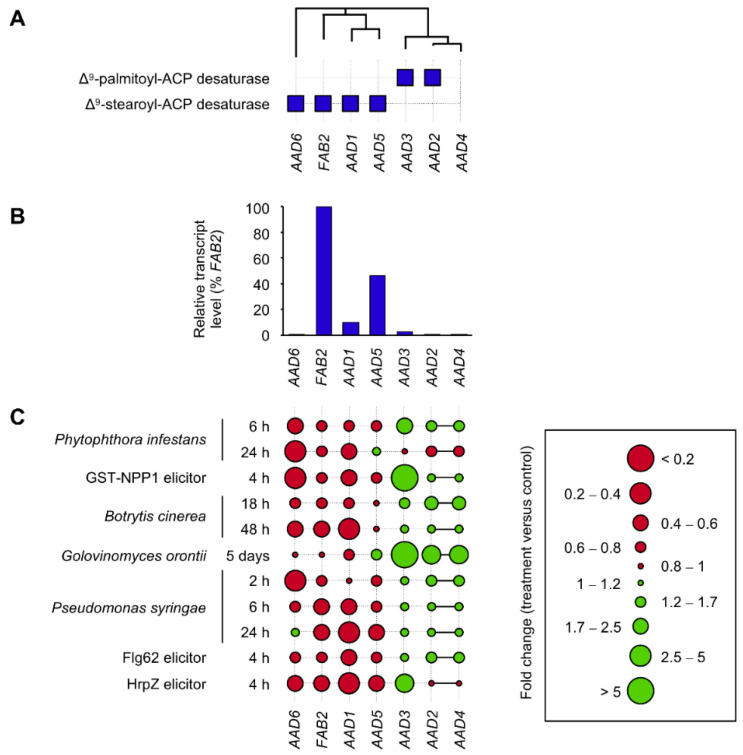
Transcriptional regulation of *AAD* genes in the leaves of *Arabidopsis thaliana* in response to biotic stress. (**A**) Phylogram, with branch lengths in arbitrary units, using the alignment of the seven *A. thaliana* AAD sequences (with gaps). Enzymatic activities of the AAD isoforms are indicated below the phylogram (blue squares). The activity of AAD4 remains unknown. (**B**) Relative *AAD* transcript levels in control leaves of *A. thaliana* expressed as a percentage of *FAB2* transcript levels. Expression levels were calculated using the data displayed on the Arabidopsis eFP Browser [57]. (**C**) Variations in *AAD* transcript levels in leaves of *A. thaliana* in response to various pathogens and elicitors and expressed in fold changes with respect to control. Bold lines between *AAD2* and *AAD4* reflect the impossibility of distinguishing between *AAD2* and *AAD4* transcripts using the Affymetrix technology due to the very similar sequences of these transcripts. Flg62, bacterial derived elicitor; GST-NPP1, oomycete-derived elicitor; HrpZ, bacterial derived elicitor.

Twenty years ago, identification by forward-genetic approaches of a first AAD-coding gene in *A. thaliana* [45] coincided with the release of the complete genome sequence of the model species [46]. It appeared that *FATTY ACID BIOSYNTHESIS2* (*FAB2*)/*SUPPRESSOR OF SALICYLIC ACID INSENSITIVE2* (*SSI2*) was part of larger multigene family comprising seven members (Figure 1A) [27]. *FAB2*, *AAD1*, *AAD5*, and *AAD6* encode Δ^9^-stearoyl–ACP desaturases [27,47,48,49] while AAD2 and AAD3 are divergent Δ^9^-palmitoyl–ACP desaturase isoforms [29,30]. To date, the enzymatic function of AAD4 remains unclear [27]. In vegetative organs, *FAB2* exhibits the highest expression levels (Figure 1B) [49] and the corresponding enzyme is usually regarded as the main Δ^9^-stearoyl–ACP desaturase isoform in this species. After the release of the *A. thaliana* genome sequence, the exploration of microarray data has quickly become a vital aspect of post-genomic research in the field. Large-scale transcriptome analyses indicate that metabolic pathways are frequently influenced by the presence of pathogens [50]. The Arabidopsis eFP Browser, which displays microarray data from Affymetrix [51], allows viewing of the expression levels of approximately 22,000 genes in the leaves of plants exposed to various pathogens such as *Phytophthora infestans* and oomycete-derived elicitors, *Botrytis cinerea*, *Golovinomyces orontii*, *Pseudomonas syringae*, and bacterial-derived elicitors, among others (Figure 1C). The exploration of the *AAD* expression profiles among the biotic stress series suggests that expression of several *AAD* genes respond to stress. Interestingly, while genes encoding Δ^9^-stearoyl–ACP desaturases isoforms appear to be essentially repressed, an opposite trend was observed for Δ^9^-palmitoyl–ACP desaturase-coding genes. When performing a large-scale transcriptional data analysis based on expression samples from Gene Expression Omnibus [52] and ArrayExpress [53], Jiang and colleagues [50] also showed that *FAB2* was significantly repressed in *A. thaliana* leaves exposed to *B. cinerea*, *Blumeria graminis* [54], *Cabbage leaf curl virus* (CaLCuV) [55], *G. orontii*, *P. syringae*, and *Sclerotinia sclerotinium*. More recently, Mine and colleagues [56] conducted an RNA-sequencing analysis of plants treated with ETI-triggering avirulent strain of *P. syringae* to gain insights into the gene regulatory networks controlling transcriptional reprogramming during ETI. A coexpression network analysis was implemented, which allowed clustering coexpressed genes into modules. This approach revealed that *FAB2* belongs to a module that gathers genes exhibiting strong and statistically significant transcriptional decrease in early hours after infection, whereas *AAD3* belongs to a module enriched for genes associated with immunity-related GO terms with strong transcriptional induction at early hours. Interestingly, using wild-type plants and mutants deficient in components of the signaling network upon challenge with virulent or ETI-triggering avirulent strains of *P. syringae*, the authors then showed that susceptible plants exhibit almost identical transcriptional responses to resistant plants, although with several hours delay.

The necrotrophic pathogen *B. cinerea* is endemic throughout the world and causes severe pre- and post-harvest losses in many crops [58]. Using the *A. thaliana*-*B. cinerea* pathosystem to investigate how the host’s defense system functions against genetic variation in the pathogen, Zhang and colleagues [58] measured defense-related phenotypes and transcriptomic responses in *A. thaliana* challenged with 96 diverse *B. cinerea* isolates. This study failed to identify any singular gene or subset of genes highly correlated with resistance and lesion areas, supporting the perspective that host resistance to *B. Cinerea* is highly polygenic. It should be noted, however, that a negative correlation between *FAB2* expression and lesion size was observed [58]. To further characterize this pathosystem and its genetic interactions dominated by complex small-effect loci displaying a high degree of interaction between the host and pathogen, a cotranscriptome study with simultaneous analysis of the host and pathogen’s transcripts was recently performed through single-sample RNA-sequencing. In addition, a GWA analysis of both host and pathogen transcriptomes was implemented to identify loci in the *B. Cinerea* genome that may affect the transcriptomes of either or both organisms [58,59,60]. This analysis revealed mostly small-effect polymorphisms dispersed throughout the pathogen genome, with several *B. cinerea trans*-eQTL hotspot loci associated with specific host or pathogen transcript coexpression modules and variation in lesion size. These loci exhibit regulatory potential in controlling the plant–pathogen interaction via modulation of gene expression to influence the lesions outcome. Several of these hotspots have an overrepresentation of photosynthesis-related functions within their targets, in good agreement with the previously characterized downregulation of photosynthesis transcripts described as a hallmark of plant immune processes [61]. Interestingly, *FAB2* was identified among the genes targeted by the *B. cinerea trans*-eQTL hotspot 10_2268522 whose nearest gene, *Bcin10g05900*, encodes a winged helix-turn-helix transcription factor [60]. Unfortunately, the molecular mechanisms underpinning the transcriptional control of *AAD* genes in response to pathogen attacks remain totally unknown. The only transcription factors known to regulate the expression of gene members of the *AAD* family, WRINKLED1 and MYB115-MYB118, participate in the developmental control of seed metabolism [30,49]. So far, these *trans*-acting factors have never been associated with stress signaling, suggesting the existence of alternative regulatory mechanisms affecting the expression of *AAD* genes in response to biotic stress.

## 4. Δ^9^-Stearoyl–ACP Desaturases and Plant–Pathogen Interactions

**Figure 2:**
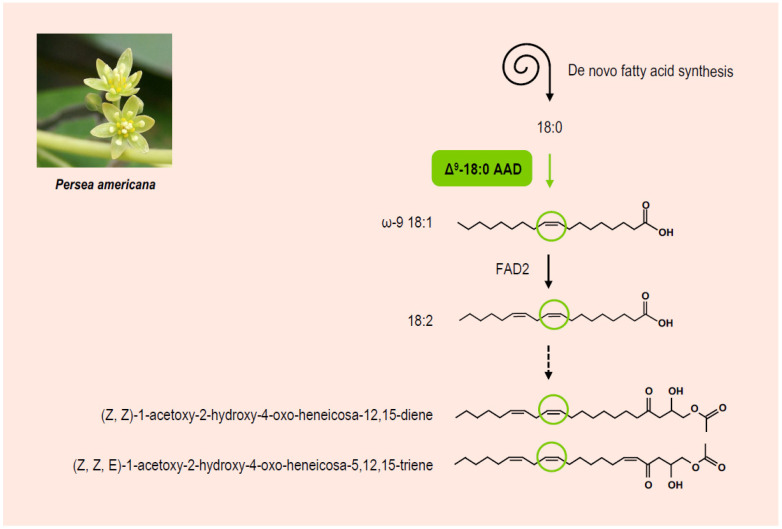
Biosynthesis of antifungal dienes in *Persea americana*. Δ^9^-18:0 AAD, Δ^9^-stearoyl–acyl carrier protein desaturase; FAD2, fatty acid desaturase 2. Picture credit: [66].

In several subtropical fruits, preformed biologically active phytochemicals have been associated with fruit resistance to pathogen attack [62]. In *Persea americana* (avocado) fruits, for example, *Colletotrichum gloeosporioides* initiates its attack by conidium germination and appressorium formation, followed by the penetration of infection hyphae into epidermal cells [63]. It has been proposed that the presence of preformed antifungal compounds such as (*Z*,*Z*)-1-acetoxy-2-hydroxy-4-oxo-heneicosa-12,15-diene and (*Z*,*Z*,*E*)-1-acetoxy-2-hydroxy-4-oxo-heneicosa-5,12,15-triene in the fruit peel regulates fruit resistance [64]. If the biosynthetic pathway for these compounds has never been fully elucidated, production of ω-9 18:1, followed by its desaturation, is thought to yield the starting compound 18:2, which is further elongated by conventional two-carbon extension (Figure 2). Interestingly, expression of an avocado gene encoding a Δ^9^-stearoyl–ACP desaturase was enhanced by different stresses including treatment with ethylene and inoculation by *C. gloeosporioides*. Furthermore, this transcriptional activation was associated with increased 18:2 levels, higher incorporation of 14C-linoleate into the antifungal dienes, and improved resistance to *C. gloeosporioides* [65], suggesting that Δ^9^-stearoyl–ACP desaturase activity not only participates in the pathway leading to antifungal diene formation, but also regulates this pathway.

In *A. thaliana*, crown galls induced by *Agrobacterium tumefaciens* encounter hypoxia, drought stress, and ROS during their development [67]. Tolerance of plant cells for that type of stress was previously shown to be associated with their capacity to maintain or increase fatty acid unsaturation of polar lipids [68] and, accordingly, levels of lipids containing 18:3 are significantly higher in tumors compared with reference stems, despite the limited oxygen availability in crown galls [48]. Two genes encoding the Δ^9^-stearoyl–ACP desaturase AAD6 and the fatty acid desaturase FAD3, respectively, were shown to be strongly expressed in tumors and proposed to participate in increasing the levels of unsaturated fatty acids. Transcriptional activation of *AAD6* is thought to be driven by hypoxia conditions associated with crown gall development [48]. However, beyond the correlation observed between *AAD6* expression and increased levels of unsaturated fatty acids in tumors, functional evidence for a role of this desaturase in stress tolerance is still lacking.

**Figure 3:**
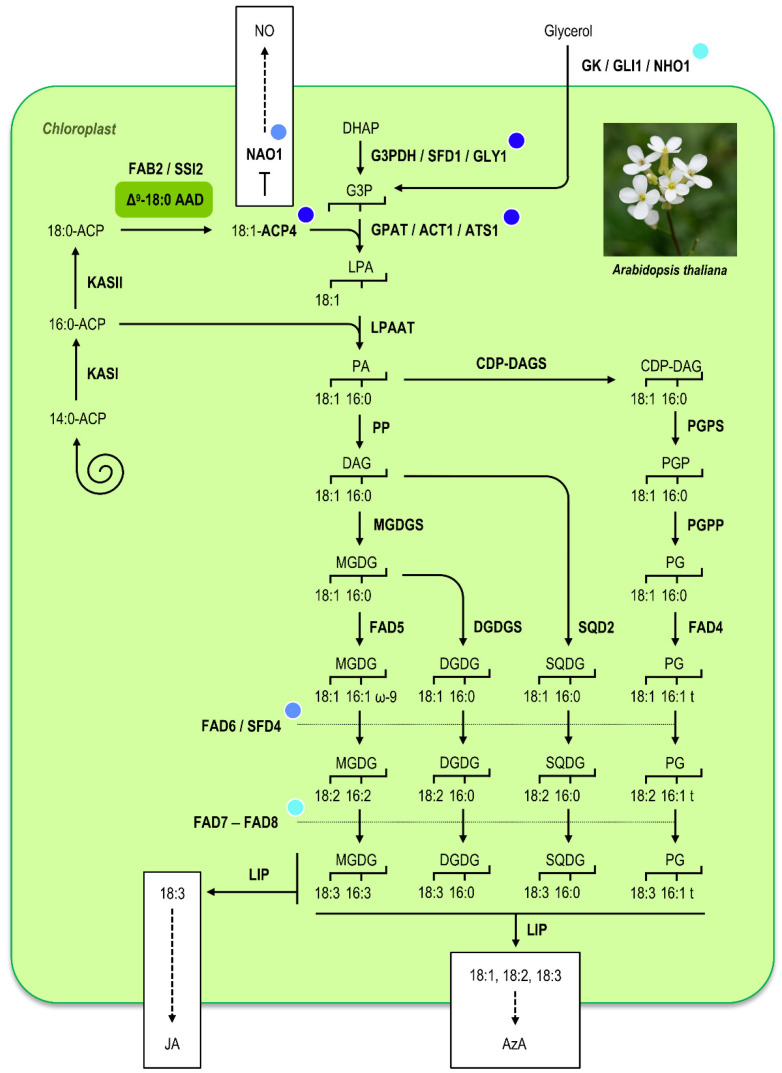
Lipid metabolism in the chloroplasts of *Arabidopsis thaliana*. The scheme depicts the different pathways involved in the biosynthesis of fatty acids and the elaboration of membrane lipids in the chloroplasts of *A. thaliana*. For the sake of clarity, export of fatty acids from the plastids and the eukaryotic lipid metabolic pathway have been omitted. Production of oleic acid by the Δ^9^-stearoyl–ACP desaturase FAB2/SSI2 is highlighted in green. Blue-colored circles denote enzymatic steps whose blockage (by mutation of corresponding genes) complements the *fab2*/*ssi2* mutant phenotype: darker shades of blue denote full phenotypic reversion while lighter shades of blue denote partial phenotypic reversion. Lipid-derived signaling molecules (JA and AzA) and NO, whose biosynthesis is regulated by oleic acid, are presented on a white background. AAD, acyl–acyl carrier protein desaturase; ACP, acyl carrier protein; AzA, azelaic acid; CDP-DAG, cytidinediphosphate-diacylglycerol; DAG, diacylglycerol; CDP-DAGS, cytidinediphosphate-diacylglycerol synthase; DHAP, dihydroxyacetone phosphate; DGDG, digalactosyldiacylglycerol; DGDGS, digalactosyldiacylglycerol synthase; FAD, fatty acid desaturase; G3P, glycerol-3-phosphate; G3PDH, glycerol-3-phosphate dehydrogenase; GK, glycerol kinase; GPAT, glycerol-3-phosphate acyltransferase; JA, jasmonic acid; KAS, fatty acid synthase complex comprising 3-ketoacyl-ACP synthase; LIP, lipase; LPA, lysophosphatidic acid; LPAAT, 1-acylglycerol-3-phosphate acyltransferase; MGDG, monogalactosyldiacylglycerol; MGDGS, monogalactosyldiacylglycerol synthase; NAO1, NITRIC OXID ASSOCIATED1; NO, nitric oxide; PA, phosphatidic acid; PG, phosphatidylglycerol; PGP, phosphatidylglycerol-phosphate; PGPP, phosphatidylglycerol-phosphate phosphatase; PGPS, phosphatidylglycerol-phosphate synthase; PP, phosphatidate phosphatase; SQD2, UDP-sulfoquinovose:diacylglycerol sulfoquinovosyltransferase; SQDG, sulfoquinovosyldiacylglycerol. Picture credit: [81].

While the above examples illustrate how ω-9 18:1 produced by Δ^9^-stearoyl–ACP desaturases can serve as a precursor for the biosynthesis of biochemical compounds playing key roles in plant–pathogen interactions, the involvement of ω-9 18:1 itself in plant immunity against pathogen infection has been disclosed by a mutant of *A. thaliana* deficient in FAB2, the major Δ^9^-stearoyl–ACP desaturase isoform in this species [45] (Figure 3). Plant response to pathogen infection relies on activation of a signal transduction pathway leading to HR and SAR. Accumulation of salicylic acid (SA) and expression of *PATHOGENESIS-RELATED* (*PR*) genes, some of which encode proteins exhibiting antimicrobial activities, correlate with the appearance of HR and SAR. The *AtNPR1* gene is a component of the SA signal transduction pathway participating in the induction of *PR* genes. The *ssi2* (*suppressor of SA insensitivity 2*) mutant was identified in a genetic screen for mutants restoring SA signaling in the *npr1* genetic background [69], and the corresponding mutation appeared to affect the Δ^9^-stearoyl–ACP desaturase-coding gene *FAB2* [45]. Among the phenotypes of *fab2*/*ssi2* mutants are dwarfing, accumulation of high levels of SA, constitutive activation of defense signaling, and spontaneous cell death. These characteristics confer broad spectrum disease resistance to multiple pathogens such as *P. syringae*, *Peronospora parasitica*, *Golovinomyces cichoracearum*, *Turnip crinkle virus* (TCV), and *Cucumber mosaic virus* (CMV) [26,70,71,72]. However, *A. thaliana fab2/ssi2* mutant plants are also defective in jasmonic acid (JA)-regulated signaling and thereby are hypersusceptible to necrotrophic pathogens like *B. cinerea* [45]. This is consistent with the fact that SA-mediated signaling usually participates in defense against biotrophs, while defense to many necrotrophic pathogens requires JA-derived signaling [73]. Importantly, although FAB2/SSI2 catalyzes the initial desaturation step required for JA biosynthesis, the *fab2*/*ssi2* mutation does not alter the levels of the JA precursor 18:3 or the induced endogenous levels of JA and cannot be rescued by exogenous JA [45]. Loss or knockdown of Δ^9^-stearoyl–ACP desaturase-coding genes is also associated with similar phenotypes in other plant species. In *G. max*, for example, silencing of the *GmSACPD-A* and *GmSACPD-B* genes confers enhanced resistance to bacterial and oomycete pathogens, namely *P. syringae* and *Phytophthora sojae* [74], while suppression of *OsSSI2* in *Oryza sativa* (rice) enhances resistance to the leaf-blight bacteria *Xanthomonas oryzae* and blast fungus *Magnaporthe grisea* [75].

The *fab2*/*ssi2* mutation confers high 18:0 level and reduced ω-9 18:1 level. Identification and characterization of *fab2*/*ssi2* suppressor mutants showed that the altered defense phenotype observed in *fab2*/*ssi2* mutant plants is due to the reduction in ω-9 18:1 level in the chloroplast (Figure 3). As a matter of fact, mutations restoring levels of plastid ω-9 18:1 in the *fab2*/*ssi2* background while maintaining high levels of 18:0 also restore wild-type phenotypes [5]. For example, a loss-of-function mutation affecting the *ACT1* gene, which encodes a glycerol-3-phosphate acyltransferase catalyzing the acylation of glycerol-3-phosphate with ω-9 18:1 [76], restores wild-type levels of ω-9 18:1 and completely reverses SA- and JA-mediated phenotypes in *fab2*/*ssi2* [77]. A similar phenotypic reversion was obtained with a loss-of-function mutation of *ACP4* gene, which inhibits the ACT1-catalyzed reaction because ω-9 18:1-ACP4 is the preferred substrate for ACT1 [78]. A mutation affecting the glycerol-3-phosphate dehydrogenase-coding gene *GLY1*/*SFD1*, which disrupts the generation of glycerol-3-phosphate from dihydroxyacetone phosphate and thereby the ACT1-catalyzed reaction, also restores a wild-type phenotype [5,79]. Conversely, exogenous application of glycerol on wild-type plants increases endogenous levels of glycerol-3-phosphate and activates the prokaryotic pathway of glycerolipid biosynthesis, resulting in a reduction in plastid ω-9 18:1 level and *fab2*/*ssi2*-like phenotypes [80]. Since the ACT1-catalyzed reaction is rate limiting, the quenching of ω-9 18:1 is even more efficient in glycerol-treated *ACT1* over-expressing lines.

It was later proposed that plastid ω-9 18:1 regulates defense signaling via suppressing NO production [82]. In *A. thaliana*, at least two routes for pathogen-responsive NO production have been described, one via the reduction of nitrate by the nitrate reductases NIA1 and NIA2, and the other via an unknown mechanism involving NITRIC OXIDE ASSOCIATED1 (NAO1), a protein bearing GTPase activity. In chloroplasts, ω-9 18:1 physically interacts with NAO1, hence repressing the GTPase activity of NAO1, in turn leading to its degradation in a protease-dependent manner. As a consequence, decreased ω-9 18:1 content stabilizes NAO1 and stimulates NO production. Furthermore, expression of *NIA1* and *NIA2* appears to be upregulated under low ω-9 18:1 condition, contributing to NO accumulation and further launching of downstream signaling, resulting in the reprogrammation of the immune response, with SA- and JA-signaling pathways being affected. Notably, the altered defense signaling in *fab2*/*ssi2* mutant plants can be partially restored by a mutation in *NAO1* and completely restored by double mutations in *NAO1* and in either of the two *NIA* genes. Since the *fab2*/*ssi2 nao1* double mutant retains *fab2*/*ssi2*-like levels of ω-9 18:1 while exhibiting partial phenotypic reversion, NOA1 was proposed to function downstream of ω-9 18:1 and to provide a direct mechanistic link between membrane characteristics and transcriptional regulation of plant defense responses [12,82]. Interestingly, ω-9 18:1 also inhibits NO synthase activity in humans [83].

However, many unknowns remain concerning the molecular mechanisms underpinning this defense response. First, it is not clear how pathogen attacks alter ω-9 18:1 level in plastids of wild-type plants, if at all. Then, the complex and intricated signaling pathways lying downstream of ω-9 18:1/NOA1 have not been fully elucidated despite the identification of several actors participating in this regulatory network including the signaling component ENHANCED DISEASE SUSCEPTIBILITY1 (EDS1) [84] and the WORKY transcription factors WRKY50 and WRKY51 [73]. EDS1 functions redundantly with SA to modulate resistance to biotrophic pathogens under low ω-9 18:1 condition, but do not participate in the repression of JA signaling, whereas WRKY50 and WRKY51 are both required for SA accumulation and the suppression of JA-responsive induction in a *fab2*/*ssi2* background. Finally, it remains unclear why partial phenotypic reversion was observed in the *fab2*/*ssi2 fad6* double mutant [5,77] and in the *fab2*/*ssi2 fad7 fad8* triple mutant [85] while the *fab2*/*ssi2 fad5* double mutant exhibited a slightly aggravated phenotype [85]. These contrasting observations suggest the existence of a complex interplay between the low ω-9 18:1-triggered signaling cascade and other lipid-derived signals originating from plastid galactolipids. Recent advances in the characterization of SAR have, for instance, revealed that NO and ROS, which serve as inducers of SAR in a concentration-dependent manner [86], triggered the generation of azelaic acid, a signaling molecule derived from the hydrolysis of unsaturated C18 fatty acids on galactolipids [10] and behaving as a chemical inducer of SAR together with SA and glycerol-3-phosphate [87].

## 5. Synthesis of ω-Anacardic Acids and Resistance to Pests in Geranium

Fatty acid derivatives consisting of a polar aromatic ring and a hydrophobic alkyl chain constitute a class of specialized metabolites known as phenolic lipids. Among them, anacardic acids (2-hydroxy-6-alkylbenzoic acids) feature a salicylic acid system substituted with saturated or unsaturated alkyl chains that have 15–17 carbons at the 6-position. They are produced by a relatively limited number of plant species, mostly in the Anacardiaceae family, as in *Anacardium occidentale* (cashew) [88] and *Pistacia vera* (pistachio) [89]. These compounds are also synthesized by some species of the Ginkgoaceae (*Ginkgo biloba*) [90], Geraniaceae (*Pelargonium x horturum*) [91], Araceae (*Philodendron scandens*) [92], Schoepfiaceae (*Schoepfia californica*) [93], Violaceae (*Viola Websteri*) [94], and Myristicaceae families (*Knema elegans* and *knema hookeriana*) [95,96]. Anacardic acids have received great attention by the community of chemicobiology researchers and pharmaceutical companies due to their potent biological activity against Alzheimer’s disease [97] as well as antitumor [98], anti-inflammatory, and anti-obese activity [99]. They also display bactericide [100], fungicide [101,102], insecticide [103,104], anti-parasite [94,105,106], and molluscicide properties [99].

Despite their great potential as therapeutic molecules and crop protection products, little is known about the in-planta biosynthesis and functions of anacardic acids. The production of these specialized metabolites seems to take place in different parts of the plant depending on the species considered. In cashew, for example, anacardic acids accumulate at high levels in a viscous pericarp fluid of the nut called ‘cashew nut shell liquid’. In contrast, zonal geranium secretes anacardic acids from glandular trichomes, specialized tissues comprised of a basal stalk topped by secretary cells accumulating essential oils. Moreover, geranium is likely one of the only plants that produce anacardic acids as a single class of phenolic lipids rather than as a complex mixture of related phenolic lipid components. The biosynthesis of anacardic acids was shown to happen through the addition of six carbons to C16 and C18 fatty acid precursors [107]. Elongation of starter fatty acyl–CoA esters is probably catalyzed by type III polyketide synthase through sequential condensation of three acetate units from malonyl–CoA [108]. The polyketide intermediate thus obtained is ultimately folded to the ring system specific to anacardic acids by aldol condensation.

So far, a physiological function of anacardic acids has been determined uniquely in zonal geranium, which has evolved a multifaceted defensive mechanism against small pests like spider mites and aphids based on the secretion of anacardic acids by glandular trichomes. Two modes of actions of these specialized metabolites have been put forward [109]. The first consists in a physical entrapment of the pests: the sticky secretion impedes pest movement, thereby reducing potential feeding and oviposition behavior. The second relies on a toxic effect of anacardic acids that increases mortality and reduces fecundity of the pests. If the exact mechanisms of action underpinning this toxic effect have not been determined with certainty, anacardic acids have been ascribed an increasing number of inhibitory activities against enzymes such as acetylcholinesterase [110], prostaglandin endoperoxide synthase [111], lipoxygenase [112], tyrosinase [113], or histone acetyltransferases [114], highlighting some interesting research areas for future investigations. Tyrosinase is indeed an important molting enzyme [113], whereas prostaglandin synthase and lipoxygenase can influence insect fecundity and maturation of eggs [111]. Conversely, in some primitive insect species lacking prostaglandins, products of the lipoxygenase can play a prostaglandin role. It is also possible that anacardic acids disrupt pest feeding by making the tissue non-nutritive or non-palatable.

**Figure 4:**
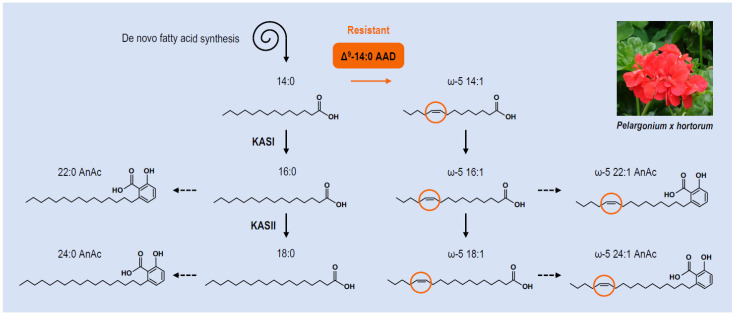
Biosynthesis of anacardic acids in *Pelargonium x hortorum*. The simplified scheme depicts the different pathways involved in the biosynthesis of fatty acids and omega-anacardic acids (AnAc) in trichomes of *Pelargonium x hortorum*. Production of myristoleic acid by Δ^9^-myristoyl–ACP desaturases in resistant genotypes is highlighted in orange. AAD, acyl–acyl carrier protein desaturase; KAS, fatty acid synthase complex comprising 3-ketoacyl–ACP synthase. Picture credit: [115].

Importantly, the effective resistance of zonal geranium to pests not only depends on the secretion of anacardic acids, but also on the chemical structure of these compounds (Figure 4). Specifically, pest resistance is mediated by the alkyl group desaturation [109]. Pest-resistant and pest-susceptible genotypes of geranium have been thoroughly characterized. The exudate from resistant plants contains approximately 90% of ω-5 monounsaturated C22 and C24 anacardic acids whereas in the susceptible genotype, the saturated 22:0 and 24:0 anacardic acids predominate, accounting for over 80% of the exudate composition [111]. These contrasted compositions result in distinct physical properties of trichome secretions. The exudate of the pest-resistant genotype is fluid under normal growth conditions, composing an efficient sticky trap, whereas the highly saturated exudate of the pest-susceptible genotype is a solid material that fails to impede pest movement. However, exudate fluidity also influences the effectiveness of the application of the potential toxin: the secretions from the resistant genotype are more effectively applied to the pest, yielding a higher level of mortality when the solid exudate of the susceptible genotype does not adhere to the exoskeleton of the pest.

The biosynthesis of ω-5 C22 and C24 anacardic acids requires a Δ^9^-myristoyl–ACP desaturase [116]. The gene encoding this desaturase is only expressed in trichomes of the pest-resistant genotype, correlating with the production of ω-5 anacardic acids and the pest-resistant phenotype. The product of the myristoyl-ACP desaturase activity, ω-5 14:1, is thought to be further elongated by the fatty acid synthase system, yielding ω-5 16:1 and ω-5 18:1 that are further metabolized through the polyketide synthase pathway. So far, the determinant of substrate specificity of the myristoyl–ACP desaturase has never been elucidated. Likewise, the identity of the transcription factor(s) regulating the transcriptional activation of the Δ^9^-myristoyl–ACP desaturase-coding gene in trichomes remains unknown. If the single dominant locus identified in geranium for conditioning ω-5 anacardic acid synthesis and subsequent pest resistance most likely corresponds to the desaturase-coding gene [117], the recalcitrance of geranium to transformation has so far prevented the confirmation of this hypothesis. The alternative hypothesis of the dominant factor encoding a transcriptional activator cannot be completely ruled out though [116].

Although anacardic acids do not have a known physiological role in most plant species producing such specialized metabolites, the wide range of bioactivity displayed by these phytochemicals suggests that they may be involved in pathogen resistance in other plants, with a mode of action distinct from the sticky trap of zonal geranium. Accumulation of anacardic acids in leaves and fruits might be related to the consumption of these tissues by pests. As a matter of fact, anacardic acids consumed as part of a diet negatively affect numerous facets of *Leptinotarsa decemlineata* (Colorado potato beetle) larval growth and development [103]. Anacardic acids therefore represent a valuable resource for integrated management in the population control of disease vectors and agricultural pests, applied as an ecofriendly chemical spray or through bioengineering production in crops. Their biological activities encourage further investigations to look for the mode of actions of these phytochemicals and for the genes encoding transcriptional regulators and biosynthetic enzymes of the anacardic acid pathway.

## 6. Synthesis of Alkenes and Attraction of Pollinators in Orchids

Orchids occur in a great variety of geographical areas and their high level of pollinator specialization is regarded as one key to their success [118]. They have evolved various cues to attract pollinators and increase efficiency in pollen delivery, which is ensured by hymenopterans like solitary bees [119]. Plant-pollinator associations are not always mutualistic and parasitism has frequently evolved on both sides [120]. Whereas two thirds of orchids reward their visitors, typically with nectar, the remaining third is enabled to produce that type of exudate and has consequently evolved alternative mechanisms based on deception. Food deception, where orchids advertise floral cues resembling those from rewarding plants, targets generalist pollinators while sexual deception, where flowers mimic the sexual signals of pollinators, attracts highly specialized pollinators [118]. The conspicuous, insect-like flowers of sexually deceptive orchids mimic both morphological characteristics and the female sex pheromones of insect pollinators, which attempt courtship or copulatory behavior with the flower and thereby remove or deliver the orchids’ pollen packets, termed pollinia [119]. Research has shown that semiochemicals were of paramount importance for pollinator attraction [121].

To elucidate the chemistry of sexual deception, pollinator perception of semiochemicals can be investigated using calcium imaging of antennal lobe activity in the pollinator brain during exposure to floral scents [122,123]. More commonly, semiochemicals can be individually tested for their ability to stimulate the pollinator’s antennal chemoreceptors using gas chromatography coupled with electroantennography [124]. A broad range of compounds pivotal for pollination has thus been discovered. Among those, known or predicted fatty acid derived semiochemicals include alkanes, alkenes, carboxylic acids, aldehydes, carboxylic esters, and chiloglottones. In many species of the Mediterranean orchid genus *Ophrys*, a blend of very long-chain alkanes and alkenes differing in carbon chain length and the position of unsaturation is the main basis for olfactory mimicry [120,125]. Bioassays have emphasized the importance of the alkene unsaturation position for controlling pollinator preference. For example, *O. sphegodes* mostly produces 9- and 12-alkenes, whereas *O. exaltata* produces high levels of 7-alkenes. The pollinator of *O. sphegodes*, *Andrena nigroaenea*, is attracted to 9- and 12-alkenes, and the addition of 7-alkenes to the odor blend reduces its attractiveness. Conversely, *Colletes cunicularius*, the pollinator of *O. exaltata*, is attracted to 7-alkenes, and the addition of 9- and 12-alkenes reduces this attraction [126].

**Figure 5:**
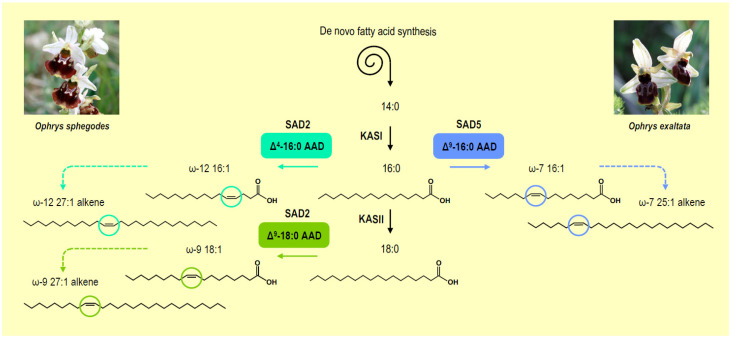
Biosynthesis of alkenes in *Ophrys sphegodes* and *Ophrys exaltata*. The simplified scheme depicts the different pathways involved in the biosynthesis of fatty acids and alkenes in the epidermal cell layer of the labellum in *Ophrys sphegodes* and *Ophrys exaltata*. Different types of acyl–acyl carrier protein desaturases with contrasted substrate specificities and regiospecificities produce different categories of monounsaturated fatty acids in the *Ophrys* species considered. As a consequence, different types of alkenes are synthesized and used as semiochemicals that attract different pollinators. AAD, acyl–acyl carrier protein desaturase; KAS, fatty acid synthase complex comprising 3-ketoacyl-ACP synthase. Picture credit: [130,131].

All currently identified *Ophrys* semiochemicals can be formed biosynthetically from fatty acid precursors. The first stage in alkane production is the de novo synthesis of C16 and C18 fatty acids in the plastid. These acyl chains are then elongated into very long-chain fatty acyl–CoAs (C20–C34) in the endoplasmic reticulum [124]. After this elongation sequence, the enzymatic complex formed by ECERIFERUM1 and ECERIFERUM3 efficiently converts very long-chain acyl–CoAs into odd-numbered very long-chain alkanes via a two-step reaction of aldehyde formation followed by decarbonylation [127,128]. The semiochemicals may then be exported just as the other components of the cuticular layer of the epidermal cells of the flower. Alkenes should follow the same biosynthetic pathway as alkanes, with the noticeable exception of an additional desaturation step potentially catalyzed by AADs prior to the elongation step [124]. In light of the essential role played by the alkene unsaturation position for governing pollinator preference, research efforts have been dedicated to identify AADs participating in alkene production in *Ophrys*. *AAD* multigene families were therefore thoroughly characterized in closely related sympatric *Ophrys* species reproductively isolated from each other due to the production of different types of alkenes and the subsequent attraction of different pollinators [126,129]. These studies have led to the discovery of specialized AAD isoforms preferentially expressed in one or the other of these species and displaying distinct enzymatic activities that could be associated with the production of different categories of alkenes (Figure 5). For example, the expression level of *SAD2* is much higher in *O. sphegodes* flowers than in *O. exaltata* flowers [126], and in vitro assays have established that *O. sphegodes* SAD2 exhibits both Δ^9^-stearoyl–ACP desaturase and Δ^4^-palmitoyl–ACP desaturase activities [129]. As such, it is very likely that SAD2 produces ω-9 18:1 and ω-12 16:1 intermediates required to build the 9- and 12-alkenes responsible for *A. nigroaenea* attraction. Conversely, SAD5 is predominant in flowers of *O. exaltata*, where this specialized Δ^9^-palmitoyl–ACP desaturase synthesizes ω-7 16:1 used for the production of 7-alkenes attracting *C. cunicularius*.

Remarkably, protein structural modeling and reconstruction of inferred ancestral state proteins have shown how the 16:0 specificity of the specialized Δ^9^-palmitoyl–ACP desaturase SAD5 is linked to just three amino acid residues lining the bottom part of its substrate-binding pocket [132]. Reconstructed ancestral state proteins at these three amino acid sites exhibit a marked preference for 18:0 substrates similar to that of the archetype Δ^9^-stearoyl–ACP desaturase. The substrate-binding pocket of the archetype desaturase is deep enough to accommodate 18:0 substrates. In contrast, changed amino acids obstruct the lower end of the substrate pocket in SAD5, leaving just enough space to accommodate a shorter 16:0 substrate with carbons 9 and 10 of the acyl chain facing the active site of the enzyme, leading to the production of ω-7 monounsaturated fatty acids. The replacement of a hydrophobic residue by a bulkier and still hydrophobic residue (as in the L145F substitution observed in SAD5) appears to be a key determinant of modified substrate specificity for all the specialized Δ^9^-palmitoyl–ACP desaturases characterized thus far [28,30]. The evolutionary trajectory of *SAD5* is proposed to have arisen by gene duplication and diversification from an ancestral ‘housekeeping’ Δ^9^-stearoyl–ACP desaturase. Modeling approaches illustrate how variations in *AAD* paralogs associated with floral scent variations can drive evolutionary divergence between orchid species, with subsequent reproductive isolation allowing for rapid sympatric speciation by pollinator shift [125]. *AAD* genes responsible for contrasted alkene production between species therefore appear as potential ‘speciation genes’, supporting the model where orchids are prime candidates for rapid pollinator-driven ‘genic’ speciation [124]. In this model, changes in a limited subset of genes with large effects do make speciation more likely than changes in many genes with small effects [119].

While the question of the molecular bases of substrate specificity has been successfully addressed for one AAD isoform in orchids, opening interesting research perspectives in the field of pollinator-driven speciation, the equally important regulatory mechanisms that differentially activate expression of the *AAD* genes remain completely unknown. The *AAD* transcripts encoding desaturases involved in alkene production are specifically accumulated in the epidermal cell layer of the labellum [132,133]. Aside from this tissue-specific accumulation pattern [134], *AAD* transcripts exhibit contrasted accumulation levels from one species to another [126]. Preliminary analyses relying on expression studies in hybrids suggest that both *cis*- and *trans*-acting factors regulate these gene expression differences. Importantly, these factors also constitute potential speciation elements that cannot be neglected.

Interestingly, recent reports have provided examples of sexual deception in the Iridaceae [135] and Asteraceae [136], suggesting that this pollination strategy may be more common than is currently known [121]. Considering both the important proportion of sex pheromones secreted by female insects to attract conspecific mates that derive from fatty acids [137] and the relatively frequent occurrence of unsaturations within the acyl chains of such compounds [138,139], it is tempting to speculate that more AADs involved in the production of semiochemicals aimed at favoring plant pollination are yet to be discovered in the plant kingdom.

## 7. Conclusions

The regio- and stereospecific unsaturation of saturated acyl chains by different types of AADs in plants provides a large pool of monounsaturated fatty acids used for the biosynthesis of different kinds of lipids and derivatives. Past research efforts have shown that a number of these molecules participate in regulatory processes and signal transduction pathways essential for plant biotic interactions. As research initiatives aimed at exploring the diversity of plant specialized metabolites become more numerous while benefiting from increasingly sophisticated and affordable-omics technologies, it is very likely that many molecules derived from unsaturated fatty acids and bearing unusual chemical functionalities will soon be discovered to participate in responses to biotic stress. As a striking example of these approaches, a pathogen-responsive gene cluster responsible for the biosynthesis of falcarindiol was recently identified in *Solanum lycopersicum* (tomato). This prototypical acetylenic lipid is derived from 18:2 and is present in edible plants like *Daucus carota* (carrot), *S. lycopersicum*, and *Apium graveolens* (celery) [140]. This dietary metabolite bearing conjugated acetylenic functionality, oxidation, and an unusual terminal vinyl group inhibits the growth of fungi as well as human cancer cell lines [141]. Future work investigating the biosynthesis, mode of action, and effectiveness of these types of compounds will be essential to better understand how plants interact with pollinators and pathogens. An improved knowledge of the underpinning biological mechanisms could be particularly beneficial for improving yields of agronomically important species and for preserving plant biodiversity in a context of climate change and modified ecological balance.
